# Bis(diphenyl-*p*-tolyl­phosphane-κ*P*)(2-hy­droxy-3,5,7-bromo­cyclo­hepta-2,4,6-trienonato-κ^2^ *O*,*O*′)copper(I)

**DOI:** 10.1107/S1600536812042286

**Published:** 2012-10-13

**Authors:** Nicola I. Barnard, Tania N. Hill

**Affiliations:** a Department of Chemistry, University of the Free State, Bloemfontein, Free State. South Africa

## Abstract

The Cu^I^ atom in the title compund, [Cu(C_7_H_2_Br_3_O_2_)(C_19_H_17_P)_2_], is located on a twofold rotation axis; the 3,5,7-tribromo­tropolonate anion coordinates as a bidentate ligand with a bite angle of 76.42 (9)°. An intra­molecular C—H⋯O inter­action occurs. Within the crystal, extensive weak C—H⋯π inter­actions contribute to the herringbone pattern observed in the packing of the mol­ecules.

## Related literature

For background to tropolone and its derivatives, see: Dewar (1945 ▶); Hill & Steyl (2008 ▶); Crous *et al.* (2005 ▶). For bis-troplolonato–copper(II) complexes, see: Chipperfield *et al.* (1998 ▶); Hasegawa *et al.* (1997 ▶); Ho (2010 ▶); Ho *et al.* (2009 ▶). For work on the effect the troplonato ligand has on the solid state and chemical behaviour of copper(I) phosphine metal complexes, see: Roodt *et al.* (2003 ▶); Steyl (2007 ▶, 2009 ▶); Steyl & Hill (2009 ▶); Steyl & Roodt (2006 ▶). 
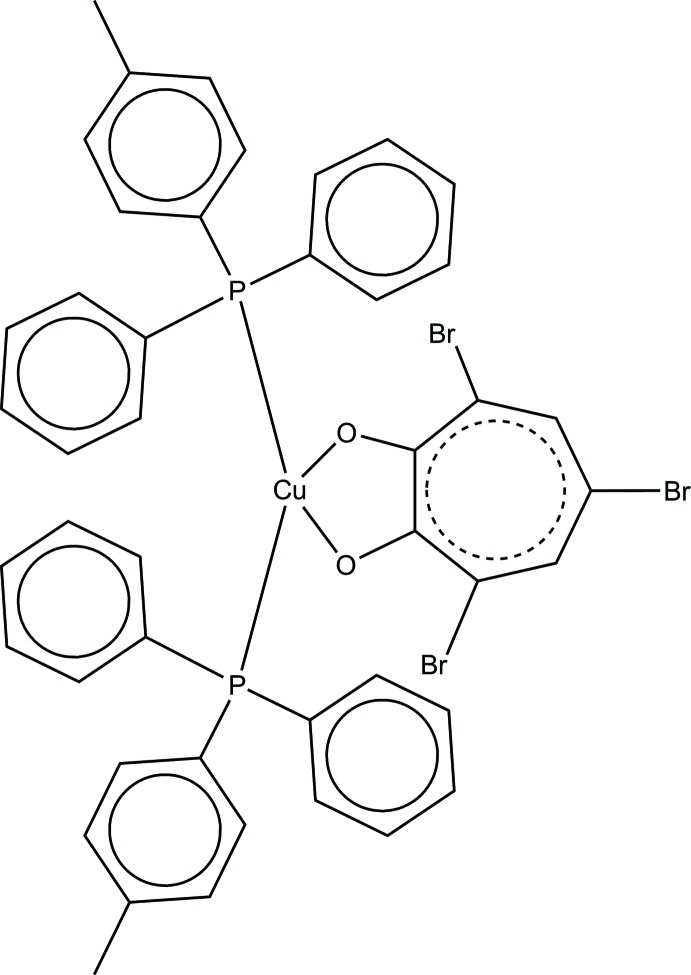


## Experimental

### 

#### Crystal data

[Cu(C_7_H_2_Br_3_O_2_)(C_19_H_17_P)_2_]
*M* *_r_* = 973.95
Monoclinic, 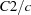

*a* = 15.4522 (8) Å
*b* = 13.9073 (8) Å
*c* = 19.3269 (10) Å
β = 103.862 (3)°
*V* = 4032.4 (4) Å^3^
*Z* = 4
Mo *K*α radiation
μ = 3.63 mm^−1^
*T* = 100 K
0.18 × 0.09 × 0.06 mm

#### Data collection

Bruker X8 APEXII 4K Kappa CCD diffractometer
Absorption correction: multi-scan (*SADABS*; Bruker, 2004 ▶) *T*_min_ = 0.686, *T*_max_ = 0.746
27602 measured reflections
5022 independent reflections
3970 reflections with *I* > 2σ(*I*)
*R*_int_ = 0.053

#### Refinement

*R*[*F* ^2^ > 2σ(*F* ^2^)] = 0.044
*wR*(*F* ^2^) = 0.130
*S* = 1.04
5022 reflections
241 parameters
H-atom parameters constrained
Δρ_max_ = 1.51 e Å^−3^
Δρ_min_ = −1.57 e Å^−3^

### 

Data collection: *APEX2* (Bruker, 2005 ▶); cell refinement: *SAINT-Plus* (Bruker, 2004 ▶); data reduction: *SAINT-Plus*; program(s) used to solve structure: *SHELXS97* (Sheldrick, 2008 ▶); program(s) used to refine structure: *SHELXL97* (Sheldrick, 2008 ▶); molecular graphics: *DIAMOND* (Brandenburg & Putz, 2005 ▶); software used to prepare material for publication: *WinGX* (Farrugia, 1999 ▶).

## Supplementary Material

Crystal structure: contains datablock(s) global, I. DOI: 10.1107/S1600536812042286/ng5299sup1.cif

Structure factors: contains datablock(s) I. DOI: 10.1107/S1600536812042286/ng5299Isup2.hkl

Additional supplementary materials:  crystallographic information; 3D view; checkCIF report

## Figures and Tables

**Table 1:** Hydrogen-bond geometry (Å, °) *Cg*2 and *Cg*3 are the centroids of the C121–C126 and C131–C136 rings, respectively.

*D*—H⋯*A*	*D*—H	H⋯*A*	*D*⋯*A*	*D*—H⋯*A*
C136—H136⋯O2	0.95	2.52	3.365 (4)	149
C115—H115⋯*Cg*3^i^	0.95	2.86	3.621 (4)	138
C137—H13*A*⋯*Cg*2^ii^	0.98	3.18	4.144 (6)	168

Symmetry codes: (i) 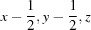; (ii) 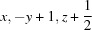.

**Fig. 1.**
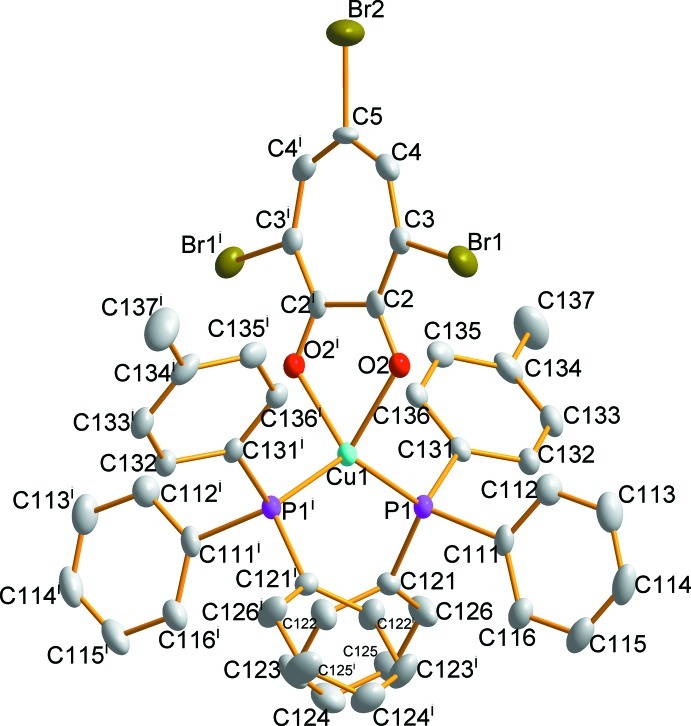
A view of (I). Displacement ellipsoids are drawn at 50% proabiity level. Hydrogen atoms have been ommited.

**Fig. 2.**
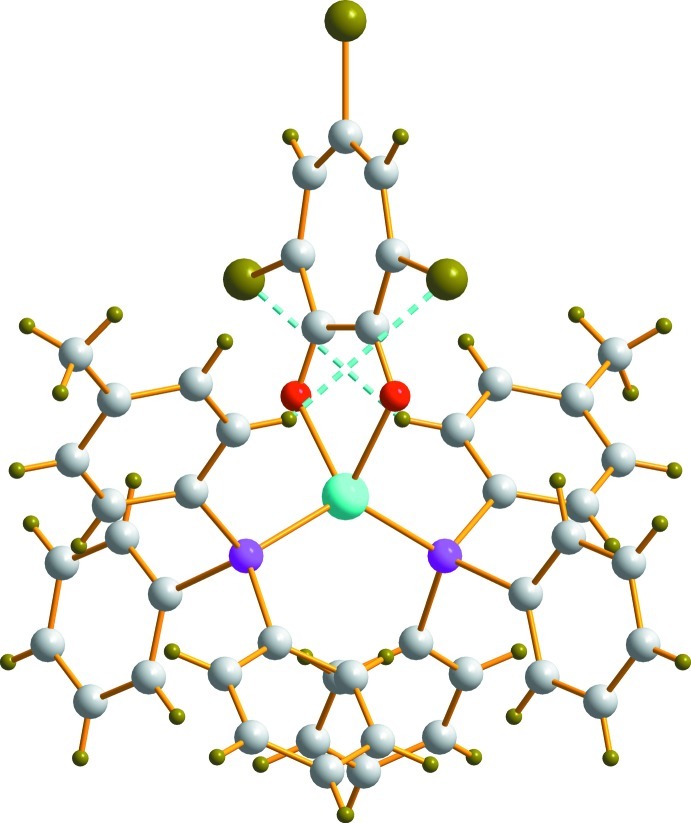
Intramolecular H···Br interactions (dashed bonds) for the title compound.

**Fig. 3.**
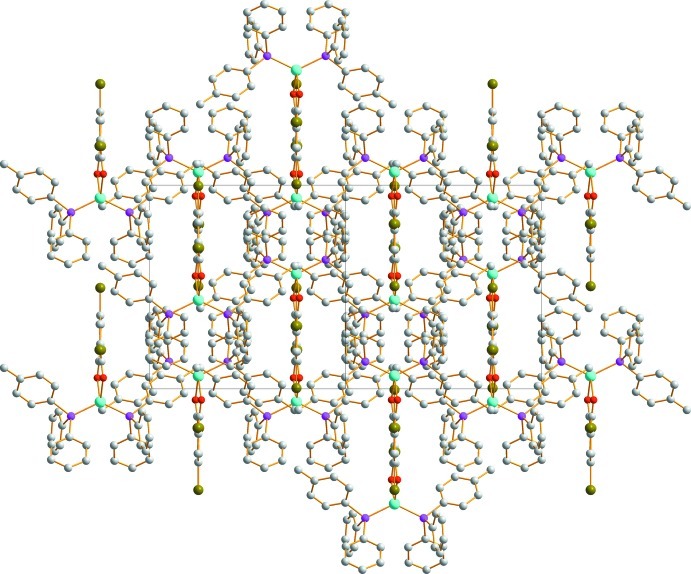
A packing diagram of the title compound, illustrating the herringbone patturnation as viewed along the [1,0,1] axis. Hydrogen atoms have been ommited.

**Fig. 4.**
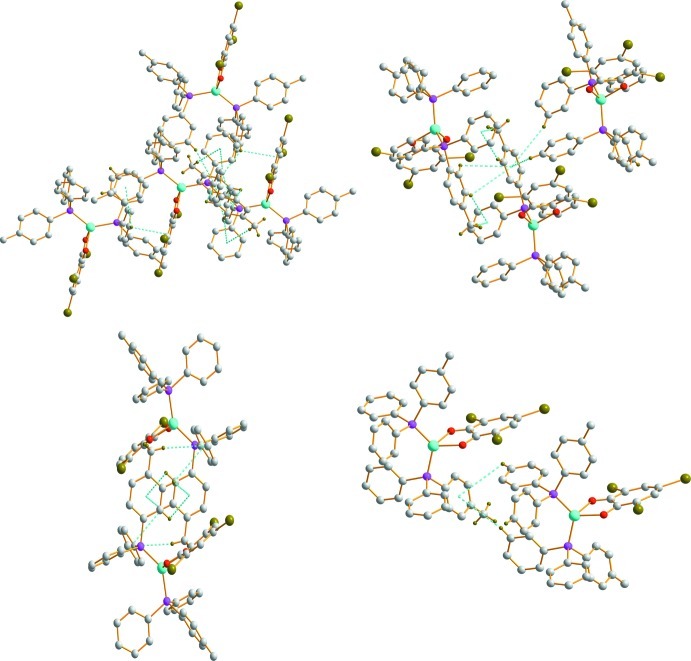
Four differing views highlighting elaborate web of H···π intermolecular interactions (dashed bonds) creating the three dimentional herringbone design, non-relevant hydrogen atoms have been ommited for clarity.

## supplementary crystallographic information

### Comment

Tropolone and its derivatives have been of interest ever since their first discovery in the early 1940's (Dewar, 1945); they are known to have applications in both pharmacology (Hill & Steyl, 2008) and catalysis (Crous *et al.*, 2005). Bis troplolonato copper(II) complexes are most frequently reported (Ho, 2010; Ho *et al.*, 2009; Chipperfield *et al.*, 1998; Hasegawa *et al.*, 1997). Recently, reseach in this area has been extended to include copper(I) phosphine metal complexes and the effect the troplonato ligand has on the solid state and chemical behaviour of these complexes (Steyl, 2007; Steyl & Roodt, 2006; Roodt *et al.*, 2003). In this paper, the structure of the tropolonato-bis[diphenyl(*p*-tolyl)-phosphine]copper(I) complex is reported (Fig. 1).

The Cu—O and Cu—P bond distances were found to be 2.090 (1) Å and 2.229 (1) Å respectively and are well within comparable ranges for copper(I) phosphine complexes. the bond angles about the Cu atom show significantly distorted tetrahedral coordination (Table 1). The bidentate bite angle O2—Cu—O2^i^ observed at 76.42 (9)° is close to analogous angles in previously reported structures (Steyl, 2009).

The title compound (I) displays intramolecular C—H···Br interactions with a distance of 3.4666 (5) Å as seen in Figure 2. Figure 3 illustrates the packing diagram for compound (I), a zigzag pattern is adopted with inverted repeating units creating diagonals in all directions. This intricate design is achieved though numerous C—H···π itermolecular interactions see Figure 4. These interactions occur between methyl H atoms of the *p*-tolyl and phenyl π, phenyl H to *p*-tolyl π, phenyl H to phenyl π and *p*-tolyl π to *p*-tolyl The C—H···π itermolecular interactions range from 3.1816 (1) Å - 3.7267 (2) Å.

### Experimental

3,5,7-Tribomotropolone (0.3 mmol) was dissolved in methanol (20 ml). To this solution was added Bis(diphenyl(*p*-tolyl)-phosphine) copper nitrate (0.3 mmol). The resulting mixture was stirred at room temperature for 30 minutes before filtering. The filtrate was then slowly evaporated yielding crystals siutable for X-ray diffraction after 48 h.

### Refinement

Hydroge atoms were placed in calculated positions, and were allowed to ride on their parent C atoms.

The final difference Fouier map had a peak/hole in the vicinity of Br1.

### Crystal data

**Table:**

[Cu(C_7_H_2_Br_3_O_2_)(C_19_H_17_P)_2_]	*F*(000) = 1944
*M**_r_* = 973.95	*D*_x_ = 1.604 Mg m^−^^3^
Monoclinic, *C*2/*c*	Mo *K*α radiation, λ = 0.71073 Å
Hall symbol: -C 2yc	Cell parameters from 8436 reflections
*a* = 15.4522 (8) Å	θ = 2.3–28.4°
*b* = 13.9073 (8) Å	µ = 3.63 mm^−^^1^
*c* = 19.3269 (10) Å	*T* = 100 K
β = 103.862 (3)°	Cuboid, green
*V* = 4032.4 (4) Å^3^	0.18 × 0.09 × 0.06 mm
*Z* = 4	

### Data collection

**Table:**

Bruker X8 APEXII 4K Kappa CCD diffractometer	5022 independent reflections
Radiation source: sealed tube	3970 reflections with *I* > 2σ(*I*)
Graphite monochromator	*R*_int_ = 0.053
Detector resolution: 512 pixels mm^-1^	θ_max_ = 28.4°, θ_min_ = 2°
φ and ω scans	*h* = −19→20
Absorption correction: multi-scan (*SADABS*; Bruker, 2004)	*k* = −15→18
*T*_min_ = 0.686, *T*_max_ = 0.746	*l* = −25→25
27602 measured reflections	

### Refinement

**Table:**

Refinement on *F*^2^	Primary atom site location: structure-invariant direct methods
Least-squares matrix: full	Secondary atom site location: difference Fourier map
*R*[*F*^2^ > 2σ(*F*^2^)] = 0.044	Hydrogen site location: inferred from neighbouring sites
*wR*(*F*^2^) = 0.130	H-atom parameters constrained
*S* = 1.04	*w* = 1/[σ^2^(*F*_o_^2^) + (0.0661*P*)^2^ + 16.6643*P*] where *P* = (*F*_o_^2^ + 2*F*_c_^2^)/3
5022 reflections	(Δ/σ)_max_ < 0.001
241 parameters	Δρ_max_ = 1.51 e Å^−^^3^
0 restraints	Δρ_min_ = −1.57 e Å^−^^3^

### Special details

**Table:**

Geometry. All s.u.'s (except the s.u. in the dihedral angle between two l.s. planes) are estimated using the full covariance matrix. The cell s.u.'s are taken into account individually in the estimation of s.u.'s in distances, angles and torsion angles; correlations between s.u.'s in cell parameters are only used when they are defined by crystal symmetry. An approximate (isotropic) treatment of cell s.u.'s is used for estimating s.u.'s involving l.s. planes.
Refinement. Refinement of *F*^2^ against ALL reflections. The weighted *R*-factor *wR* and goodness of fit *S* are based on *F*^2^, conventional *R*-factors *R* are based on *F*, with *F* set to zero for negative *F*^2^. The threshold expression of *F*^2^ > 2σ(*F*^2^) is used only for calculating *R*-factors(gt) *etc*. and is not relevant to the choice of reflections for refinement. *R*-factors based on *F*^2^ are statistically about twice as large as those based on *F*, and *R*- factors based on ALL data will be even larger.

### Fractional atomic coordinates and isotropic or equivalent isotropic displacement parameters (Å^2^)

**Table:**

	*x*	*y*	*z*	*U*_iso_*/*U*_eq_	
C2	0.5382 (2)	0.6353 (3)	0.28258 (18)	0.0213 (7)	
C3	0.5717 (2)	0.7199 (3)	0.32159 (19)	0.0230 (7)	
C4	0.5556 (2)	0.8171 (3)	0.3086 (2)	0.0294 (8)	
H4	0.5867	0.8596	0.3446	0.035*	
C5	0.5	0.8599 (3)	0.25	0.0295 (12)	
C111	0.3102 (2)	0.3211 (3)	0.25441 (19)	0.0242 (7)	
C112	0.2526 (2)	0.3880 (3)	0.2148 (2)	0.0309 (8)	
H112	0.2662	0.4546	0.2202	0.037*	
C113	0.1744 (3)	0.3578 (4)	0.1667 (2)	0.0373 (10)	
H113	0.1339	0.4041	0.1411	0.045*	
C114	0.1562 (3)	0.2615 (4)	0.1565 (2)	0.0375 (10)	
H114	0.1041	0.2411	0.1227	0.045*	
C115	0.2132 (3)	0.1946 (3)	0.1954 (2)	0.0384 (10)	
H115	0.2003	0.128	0.1883	0.046*	
C116	0.2898 (2)	0.2235 (3)	0.2451 (2)	0.0310 (8)	
H116	0.3281	0.1769	0.2725	0.037*	
C121	0.4606 (2)	0.2639 (2)	0.36831 (19)	0.0221 (7)	
C122	0.5385 (3)	0.2234 (3)	0.3588 (2)	0.0317 (8)	
H122	0.5645	0.2472	0.3223	0.038*	
C123	0.5799 (3)	0.1478 (3)	0.4021 (3)	0.0432 (11)	
H123	0.6334	0.1205	0.3949	0.052*	
C124	0.5423 (3)	0.1126 (3)	0.4559 (2)	0.0412 (10)	
H124	0.57	0.0613	0.4856	0.049*	
C125	0.4650 (3)	0.1526 (3)	0.4655 (3)	0.0470 (12)	
H125	0.4394	0.1292	0.5023	0.056*	
C126	0.4237 (3)	0.2272 (3)	0.4219 (3)	0.0391 (10)	
H126	0.3697	0.2535	0.4288	0.047*	
C131	0.3738 (2)	0.4421 (2)	0.37497 (17)	0.0202 (7)	
C132	0.2920 (2)	0.4256 (3)	0.39248 (19)	0.0236 (7)	
H132	0.2525	0.3778	0.3677	0.028*	
C133	0.2690 (2)	0.4791 (3)	0.4460 (2)	0.0277 (8)	
H133	0.2138	0.4667	0.4579	0.033*	
C134	0.3244 (3)	0.5498 (3)	0.4823 (2)	0.0303 (8)	
C135	0.4049 (2)	0.5684 (3)	0.46311 (19)	0.0263 (7)	
H135	0.443	0.6183	0.4864	0.032*	
C136	0.4292 (2)	0.5143 (3)	0.41023 (18)	0.0222 (7)	
H136	0.4841	0.527	0.3981	0.027*	
C137	0.3016 (3)	0.6032 (4)	0.5422 (3)	0.0512 (13)	
H13A	0.3485	0.6502	0.5612	0.077*	
H13B	0.2964	0.5579	0.5798	0.077*	
H13C	0.2447	0.6368	0.5249	0.077*	
O2	0.56859 (15)	0.55420 (17)	0.30362 (13)	0.0227 (5)	
P1	0.41329 (5)	0.36611 (6)	0.31230 (5)	0.01920 (18)	
Cu1	0.5	0.43613 (4)	0.25	0.01987 (15)	
Br1	0.65536 (3)	0.69166 (3)	0.40942 (2)	0.03295 (13)	
Br2	0.5	0.99600 (5)	0.25	0.0638 (3)	

### Atomic displacement parameters (Å^2^)

**Table:**

	*U*^11^	*U*^22^	*U*^33^	*U*^12^	*U*^13^	*U*^23^
C2	0.0140 (14)	0.0293 (18)	0.0231 (17)	−0.0008 (13)	0.0096 (13)	0.0021 (14)
C3	0.0171 (14)	0.0279 (18)	0.0259 (17)	−0.0019 (13)	0.0090 (13)	−0.0002 (14)
C4	0.0225 (17)	0.0277 (19)	0.041 (2)	−0.0066 (14)	0.0132 (15)	−0.0078 (16)
C5	0.027 (2)	0.011 (2)	0.054 (3)	0	0.016 (2)	0
C111	0.0150 (14)	0.036 (2)	0.0230 (17)	−0.0030 (14)	0.0071 (12)	−0.0048 (15)
C112	0.0273 (18)	0.037 (2)	0.0276 (19)	−0.0005 (16)	0.0047 (15)	−0.0003 (16)
C113	0.0256 (19)	0.061 (3)	0.0243 (19)	0.0036 (18)	0.0034 (15)	0.0005 (18)
C114	0.0222 (17)	0.062 (3)	0.030 (2)	−0.0106 (18)	0.0086 (15)	−0.015 (2)
C115	0.030 (2)	0.047 (3)	0.041 (2)	−0.0167 (18)	0.0150 (18)	−0.017 (2)
C116	0.0234 (17)	0.036 (2)	0.036 (2)	−0.0069 (15)	0.0102 (15)	−0.0061 (17)
C121	0.0212 (15)	0.0196 (17)	0.0260 (17)	0.0014 (13)	0.0070 (13)	−0.0005 (13)
C122	0.0262 (18)	0.036 (2)	0.036 (2)	0.0055 (16)	0.0138 (16)	0.0070 (17)
C123	0.034 (2)	0.044 (3)	0.055 (3)	0.0189 (19)	0.018 (2)	0.018 (2)
C124	0.045 (2)	0.034 (2)	0.047 (2)	0.0110 (19)	0.015 (2)	0.0116 (19)
C125	0.054 (3)	0.040 (2)	0.057 (3)	0.013 (2)	0.034 (2)	0.021 (2)
C126	0.035 (2)	0.036 (2)	0.055 (3)	0.0110 (17)	0.027 (2)	0.015 (2)
C131	0.0173 (14)	0.0229 (17)	0.0209 (16)	0.0055 (13)	0.0057 (12)	0.0038 (13)
C132	0.0178 (15)	0.0269 (17)	0.0273 (18)	0.0018 (13)	0.0076 (13)	0.0003 (14)
C133	0.0203 (16)	0.037 (2)	0.0273 (18)	0.0075 (15)	0.0077 (14)	0.0022 (15)
C134	0.0307 (18)	0.036 (2)	0.0238 (18)	0.0155 (16)	0.0051 (14)	−0.0003 (16)
C135	0.0262 (17)	0.0257 (18)	0.0227 (17)	0.0044 (14)	−0.0026 (13)	0.0007 (14)
C136	0.0176 (14)	0.0236 (17)	0.0239 (17)	0.0037 (13)	0.0021 (12)	0.0024 (13)
C137	0.045 (3)	0.070 (3)	0.039 (2)	0.012 (2)	0.010 (2)	−0.018 (2)
O2	0.0171 (11)	0.0233 (13)	0.0272 (12)	−0.0008 (9)	0.0041 (9)	0.0014 (10)
P1	0.0145 (4)	0.0208 (4)	0.0234 (4)	−0.0004 (3)	0.0068 (3)	−0.0008 (3)
Cu1	0.0149 (3)	0.0214 (3)	0.0249 (3)	0	0.0078 (2)	0
Br1	0.0317 (2)	0.0367 (2)	0.0276 (2)	−0.00878 (16)	0.00144 (15)	−0.00242 (16)
Br2	0.0539 (4)	0.0268 (3)	0.1064 (7)	0	0.0112 (4)	0

### Geometric parameters (Å, º)

**Table:**

C2—O2	1.252 (4)	C123—C124	1.396 (6)
C2—C3	1.426 (5)	C123—H123	0.95
C2—C2^i^	1.506 (6)	C124—C125	1.370 (6)
C3—C4	1.387 (5)	C124—H124	0.95
C3—Br1	1.911 (4)	C125—C126	1.391 (6)
C4—C5	1.382 (5)	C125—H125	0.95
C4—H4	0.95	C126—H126	0.95
C5—C4^i^	1.382 (5)	C131—C136	1.388 (5)
C5—Br2	1.893 (5)	C131—C132	1.403 (4)
C111—C112	1.384 (5)	C131—P1	1.820 (3)
C111—C116	1.395 (5)	C132—C133	1.387 (5)
C111—P1	1.824 (3)	C132—H132	0.95
C112—C113	1.400 (5)	C133—C134	1.381 (6)
C112—H112	0.95	C133—H133	0.95
C113—C114	1.375 (7)	C134—C135	1.405 (5)
C113—H113	0.95	C134—C137	1.486 (6)
C114—C115	1.374 (7)	C135—C136	1.391 (5)
C114—H114	0.95	C135—H135	0.95
C115—C116	1.392 (6)	C136—H136	0.95
C115—H115	0.95	C137—H13A	0.98
C116—H116	0.95	C137—H13B	0.98
C121—C122	1.381 (5)	C137—H13C	0.98
C121—C126	1.393 (5)	O2—Cu1	2.090 (2)
C121—P1	1.830 (4)	P1—Cu1	2.2284 (9)
C122—C123	1.398 (6)	Cu1—O2^i^	2.090 (2)
C122—H122	0.95	Cu1—P1^i^	2.2284 (9)
			
O2—C2—C3	120.7 (3)	C124—C125—H125	119.7
O2—C2—C2^i^	115.45 (19)	C126—C125—H125	119.7
C3—C2—C2^i^	123.7 (2)	C125—C126—C121	120.9 (4)
C4—C3—C2	133.0 (3)	C125—C126—H126	119.5
C4—C3—Br1	114.5 (3)	C121—C126—H126	119.5
C2—C3—Br1	112.5 (3)	C136—C131—C132	119.0 (3)
C5—C4—C3	128.1 (4)	C136—C131—P1	118.8 (2)
C5—C4—H4	115.9	C132—C131—P1	122.0 (3)
C3—C4—H4	115.9	C133—C132—C131	119.9 (3)
C4^i^—C5—C4	129.0 (5)	C133—C132—H132	120.1
C4^i^—C5—Br2	115.5 (2)	C131—C132—H132	120.1
C4—C5—Br2	115.5 (2)	C134—C133—C132	121.5 (3)
C112—C111—C116	119.1 (3)	C134—C133—H133	119.2
C112—C111—P1	117.4 (3)	C132—C133—H133	119.2
C116—C111—P1	123.4 (3)	C133—C134—C135	118.5 (3)
C111—C112—C113	120.2 (4)	C133—C134—C137	121.2 (4)
C111—C112—H112	119.9	C135—C134—C137	120.3 (4)
C113—C112—H112	119.9	C136—C135—C134	120.4 (3)
C114—C113—C112	120.2 (4)	C136—C135—H135	119.8
C114—C113—H113	119.9	C134—C135—H135	119.8
C112—C113—H113	119.9	C131—C136—C135	120.6 (3)
C113—C114—C115	119.9 (4)	C131—C136—H136	119.7
C113—C114—H114	120.1	C135—C136—H136	119.7
C115—C114—H114	120.1	C134—C137—H13A	109.5
C114—C115—C116	120.6 (4)	C134—C137—H13B	109.5
C114—C115—H115	119.7	H13A—C137—H13B	109.5
C116—C115—H115	119.7	C134—C137—H13C	109.5
C115—C116—C111	119.9 (4)	H13A—C137—H13C	109.5
C115—C116—H116	120.1	H13B—C137—H13C	109.5
C111—C116—H116	120.1	C2—O2—Cu1	116.1 (2)
C122—C121—C126	118.3 (3)	C131—P1—C111	102.98 (15)
C122—C121—P1	118.4 (3)	C131—P1—C121	101.98 (16)
C126—C121—P1	123.2 (3)	C111—P1—C121	105.21 (17)
C121—C122—C123	121.0 (4)	C131—P1—Cu1	116.55 (12)
C121—C122—H122	119.5	C111—P1—Cu1	111.62 (12)
C123—C122—H122	119.5	C121—P1—Cu1	116.91 (11)
C122—C123—C124	119.9 (4)	O2—Cu1—O2^i^	76.42 (13)
C122—C123—H123	120.1	O2—Cu1—P1	112.00 (7)
C124—C123—H123	120.1	O2^i^—Cu1—P1	108.19 (7)
C125—C124—C123	119.3 (4)	O2—Cu1—P1^i^	108.19 (7)
C125—C124—H124	120.3	O2^i^—Cu1—P1^i^	112.00 (7)
C123—C124—H124	120.3	P1—Cu1—P1^i^	128.18 (5)
C124—C125—C126	120.6 (4)		
			
O2—C2—C3—C4	174.7 (4)	C134—C135—C136—C131	0.9 (5)
C2^i^—C2—C3—C4	−9.2 (7)	C3—C2—O2—Cu1	171.0 (2)
O2—C2—C3—Br1	−5.6 (4)	C2^i^—C2—O2—Cu1	−5.4 (4)
C2^i^—C2—C3—Br1	170.5 (3)	C136—C131—P1—C111	−157.1 (3)
C2—C3—C4—C5	−1.1 (6)	C132—C131—P1—C111	27.6 (3)
Br1—C3—C4—C5	179.2 (2)	C136—C131—P1—C121	94.0 (3)
C3—C4—C5—C4^i^	2.7 (3)	C132—C131—P1—C121	−81.3 (3)
C3—C4—C5—Br2	−177.3 (3)	C136—C131—P1—Cu1	−34.5 (3)
C116—C111—C112—C113	0.9 (5)	C132—C131—P1—Cu1	150.1 (2)
P1—C111—C112—C113	176.9 (3)	C112—C111—P1—C131	62.6 (3)
C111—C112—C113—C114	−2.6 (6)	C116—C111—P1—C131	−121.6 (3)
C112—C113—C114—C115	2.2 (6)	C112—C111—P1—C121	169.0 (3)
C113—C114—C115—C116	−0.1 (6)	C116—C111—P1—C121	−15.1 (3)
C114—C115—C116—C111	−1.6 (6)	C112—C111—P1—Cu1	−63.2 (3)
C112—C111—C116—C115	1.2 (5)	C116—C111—P1—Cu1	112.6 (3)
P1—C111—C116—C115	−174.6 (3)	C122—C121—P1—C131	−141.0 (3)
C126—C121—C122—C123	−0.4 (6)	C126—C121—P1—C131	36.4 (4)
P1—C121—C122—C123	177.2 (4)	C122—C121—P1—C111	111.8 (3)
C121—C122—C123—C124	−0.2 (7)	C126—C121—P1—C111	−70.8 (4)
C122—C123—C124—C125	0.1 (8)	C122—C121—P1—Cu1	−12.7 (3)
C123—C124—C125—C126	0.4 (8)	C126—C121—P1—Cu1	164.8 (3)
C124—C125—C126—C121	−1.0 (8)	C2—O2—Cu1—O2^i^	2.14 (18)
C122—C121—C126—C125	0.9 (7)	C2—O2—Cu1—P1	−102.3 (2)
P1—C121—C126—C125	−176.5 (4)	C2—O2—Cu1—P1^i^	111.2 (2)
C136—C131—C132—C133	−2.1 (5)	C131—P1—Cu1—O2	26.45 (14)
P1—C131—C132—C133	173.3 (3)	C111—P1—Cu1—O2	144.35 (15)
C131—C132—C133—C134	0.8 (6)	C121—P1—Cu1—O2	−94.47 (15)
C132—C133—C134—C135	1.3 (6)	C131—P1—Cu1—O2^i^	−55.86 (14)
C132—C133—C134—C137	−176.7 (4)	C111—P1—Cu1—O2^i^	62.04 (15)
C133—C134—C135—C136	−2.1 (5)	C121—P1—Cu1—O2^i^	−176.78 (14)
C137—C134—C135—C136	175.8 (4)	C131—P1—Cu1—P1^i^	164.68 (12)
C132—C131—C136—C135	1.2 (5)	C111—P1—Cu1—P1^i^	−77.41 (13)
P1—C131—C136—C135	−174.3 (3)	C121—P1—Cu1—P1^i^	43.76 (13)

Symmetry code: (i) −*x*+1, *y*, −*z*+1/2.

### Hydrogen-bond geometry (Å, º)

Cg2 and Cg3 are the centroids of the C121–C126 and C131–C136 rings, respectively.

**Table:**

*D*—H···*A*	*D*—H	H···*A*	*D*···*A*	*D*—H···*A*
C136—H136···O2	0.95	2.52	3.365 (4)	149
C115—H115···*Cg*3^ii^	0.95	2.86	3.621 (4)	138
C137—H13*A*···*Cg*2^iii^	0.98	3.18	4.144 (6)	168

Symmetry codes: (ii) *x*−1/2, *y*−1/2, *z*; (iii) *x*, −*y*+1, *z*+1/2.
